# Dataset for comparison between single and double pilot injection in diesel-natural gas dual fuel engine

**DOI:** 10.1016/j.dib.2019.104963

**Published:** 2019-12-10

**Authors:** Luigi De Simio, Sabato Iannaccone

**Affiliations:** Istituto Motori, National Research Council, Via Marconi 4, 80125, Napoli, Italy

**Keywords:** Double-pilot injection, Dual fuel, Natural gas, Low-temperature combustion

## Abstract

The present data article is based on the research work which investigates the low-temperature combustion (LTC) in a dual fuel light duty engine. The LTC mode was activated by means of double pilot injection to control the energy release rate in the first combustion stage, thereby minimizing the increase of the rate of pressure and allowing the operation under LTC. This data article presents all the data which supports the choice for double pilot injection vs single pilot injection for that research. In this experimental work the engine was fueled with diesel, in full-diesel (FD) mode, and in dual fuel (DF) mode with natural gas and natural gas – hydrogen mixtures as main fuel. In DF mode the pilot diesel fuel was injected both with a single and a double injection at the same engine speed and torque. The pressure cycle in one of the four cylinders, the intake manifold pressure and the injector current signal were acquired on a crank angle basis for 100 consecutive engine cycles. Analysis of combustion rate, maximum pressure rise and fuel/air flow rate were performed. The data set, which also includes some engine control parameters, the combustion chamber geometry and some injector features, can potentially be reused to numerically model the combustion phenomena and, in particular, to investigate the effect on the ignition phase of combustion in LTC, also considering the variability from cycle to cycle.

**Table:** Specifications Table

Subject	Energy Engineering and Power Technology
Specific subject area	Dual fuel internal combustion engine for generators or hybrid propulsion
Type of data	Table Image Figure
How data were acquired	Experimental analysis in engine testing laboratory
Data format	Raw Analyzed Filtered
Parameters for data collection	Engine speed and load at 2000 rpm and 100 Nm respectively. FD e DF mode fueling. In DF three pilot injection strategies: 1) double pilot injection (DPI) with minimum diesel for stable combustion, single pilot injection (SPI), both 2) with minimum diesel and 3) with the same diesel of DPI. Start of first diesel injection set to 10, 20, 25, 35 and 45 crank angle degree (CAD) before top dead center (BTDC).
Description of data collection	The cycle pressure in the cylinder, the intake manifold pressure and the injector current signal were acquired on a crank angle basis for 100 consecutive engine cycles. Analysis of combustion rate, maximum pressure rise and fuel/air flow rate were performed. The data set also includes the parameters of the engine, the combustion chamber and the diesel fuel injector.
Data source location	Istituto Motori, National Research Council Napoli Italy
Data accessibility	With the article and in a public repository Repository name: Mendeley Data Data identification number: https://doi.org/10.17632/d8fhndxmtx.1 Direct URL to data: https://data.mendeley.com/datasets/d8fhndxmtx/1
Related research article	Author's name Luigi De Simio, Sabato Iannaccone Title Gaseous and particle emissions in low-temperature combustion diesel–HCNG dual-fuel operation with double pilot injection Journal Applied Energy DOI https://doi.org/10.1016/j.apenergy.2019.113602

**Table:** 

**Value of the Data** • The data set is useful to compare double pilot injection with single pilot injection strategy • The data set can benefit researchers who want to develop new injection strategies for dual fuel engines, in particular to achieve low temperature combustion • The additional value of this data set consists in the possibility of using them also for developing numerical model, by evaluating both the average behavior and the variation of the parameters from cycle to cycle engine

## 1 Data

The data presented in this paper was based on the experimental activity which investigates the LTC in a dual fuel light duty engine [1]. The experimental analysis carried out in Ref. [1] was aimed at measure the performance and emissions of an internal combustion engine fueled with natural gas and natural gas/hydrogen mixtures as main fuels, pilot ignited by small amount of diesel fuel, in the case of LTC combustion. To activate this mode of combustion it was necessary to increase the injection advance of the pilot diesel fuel, with respect to the end of the compression stroke, in a remarkable manner. The advance pilot injection can lead to a strong increase in the maximum pressure gradient which, beyond certain limits, causes an unreliable operation of the engine. Therefore, the limit value of 15 bar/CAD was used during the tests to set the maximum injection advance increasing, as done by other researchers [2]. With this limit, it was decided to use the DPI strategy instead of the SPI, to control the energy release rate in the first combustion stage, allowing the operation under LTC. This article reports all the data which support that choice, most of which have been not reported in Ref. [1].

**Fig. 1:**
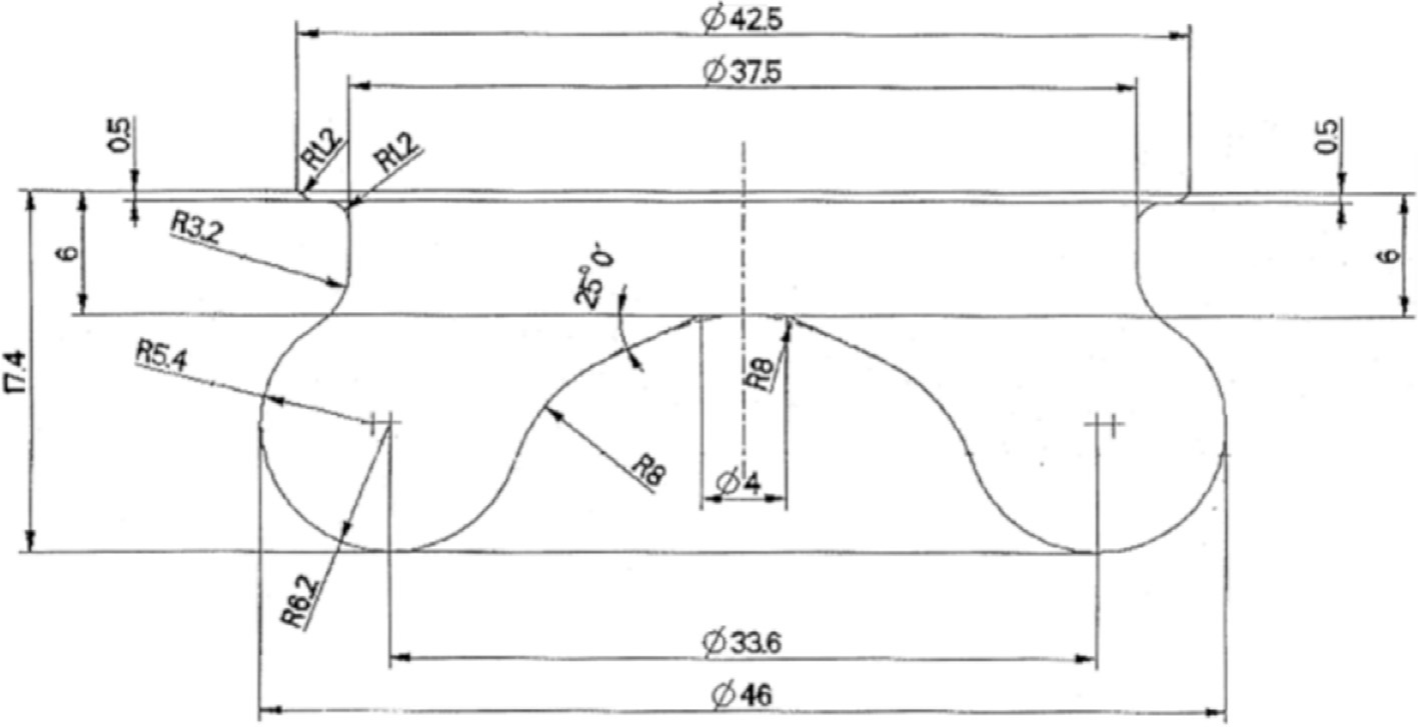
Drawing of the combustion chamber (lengths in mm).

**Fig. 2:**
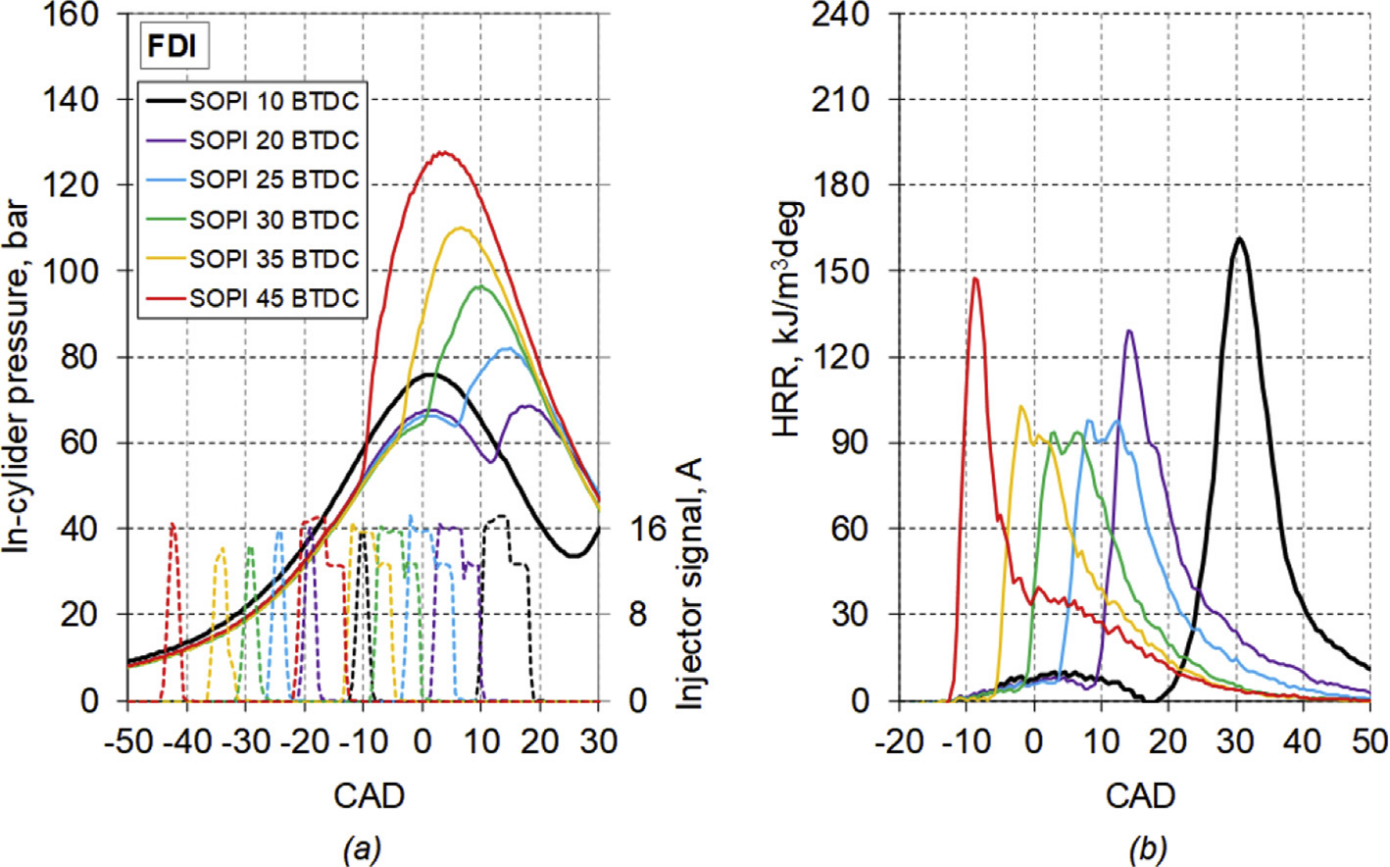
Average acquisition of *(a)* the indicated pressure cycle, diesel injector control signal and *(b)* the heat release rate curve, for the FDI case.

**Fig. 3:**
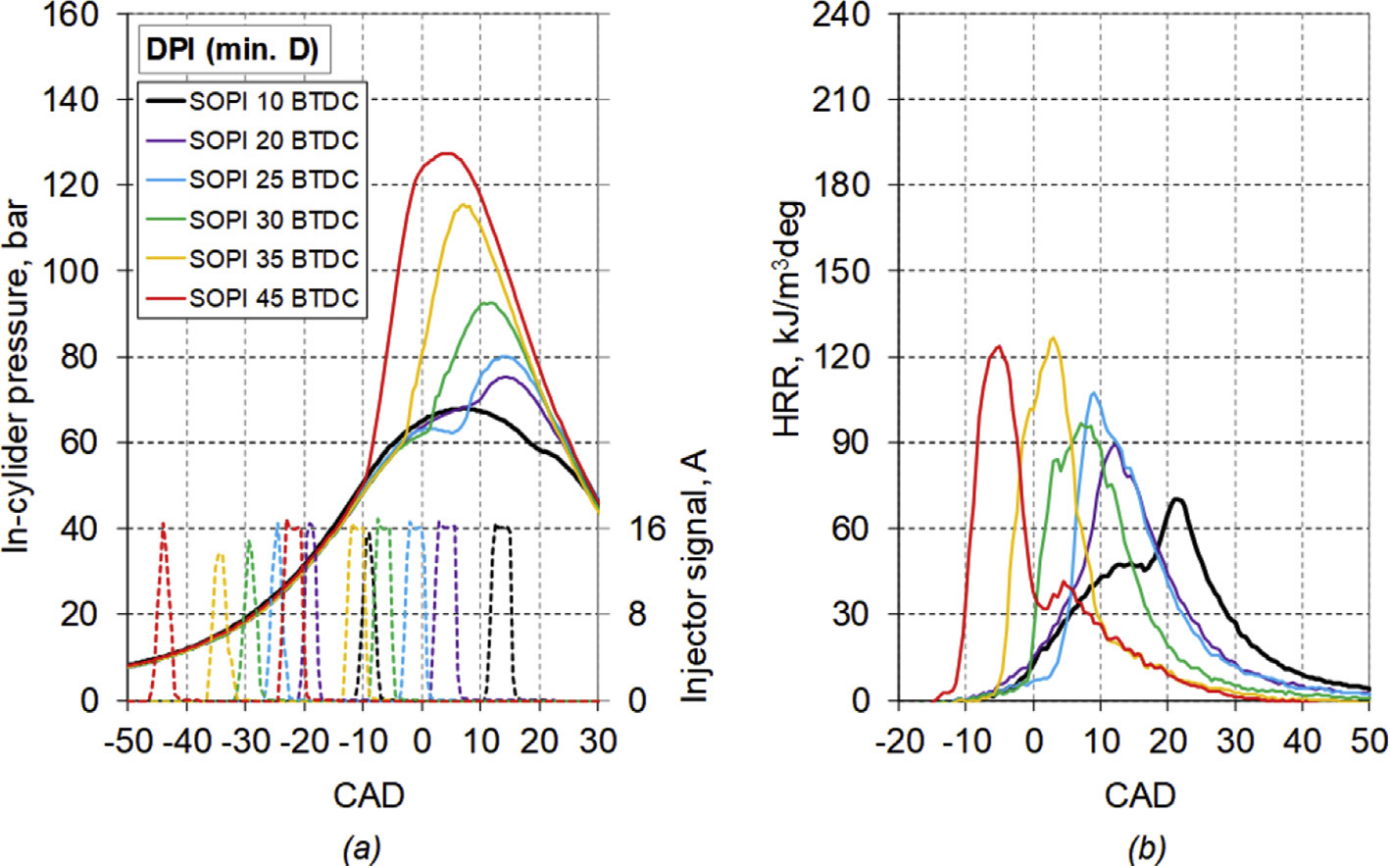
Average acquisition of *(a)* the indicated pressure cycle, diesel injector control signal and *(b)* the heat release rate curve, for the DPI case.

**Fig. 4:**
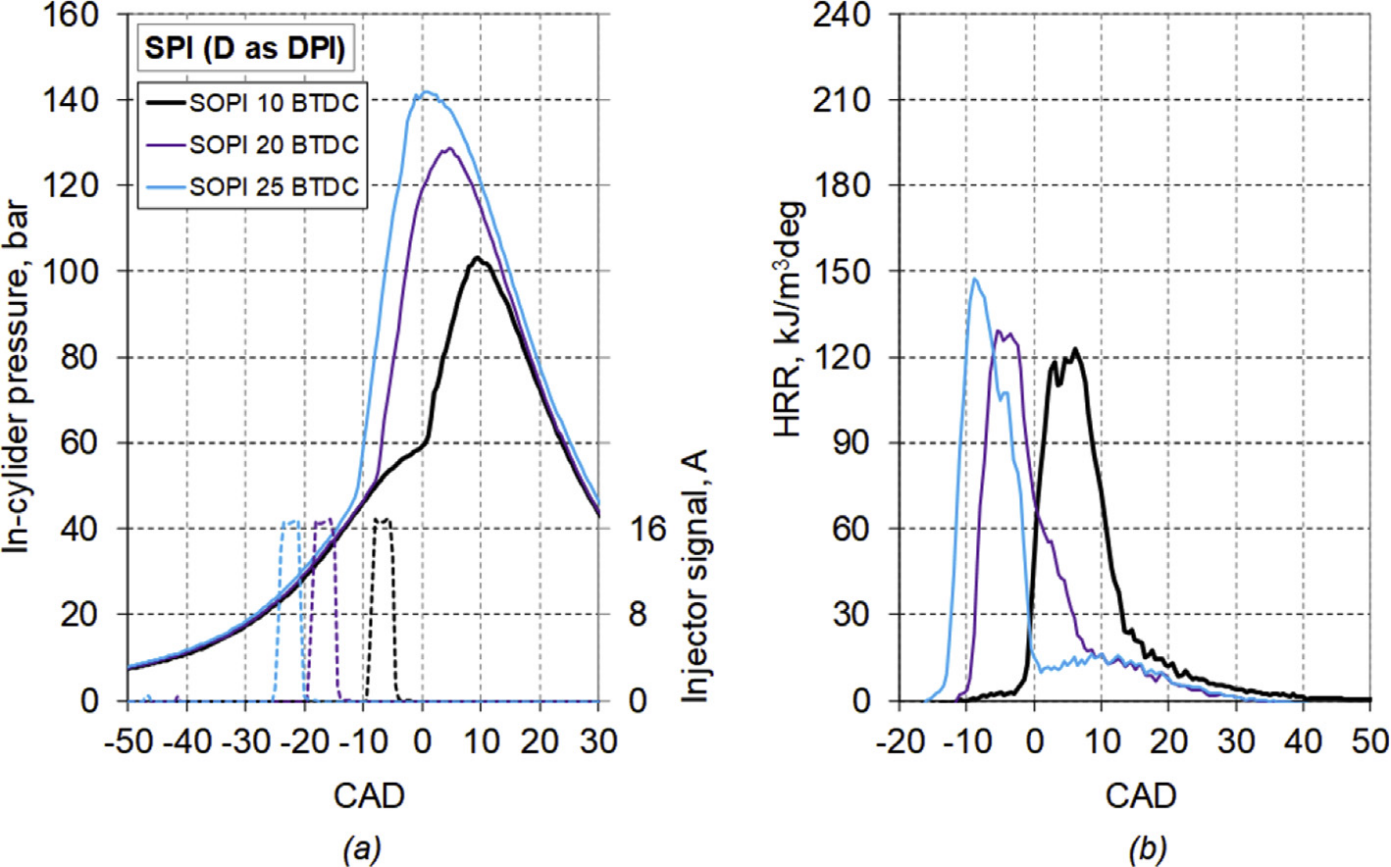
Average acquisition of *(a)* the indicated pressure cycle, diesel injector control signal and *(b)* the heat release rate curve, for the SPI (D as DPI case.

**Fig. 5:**
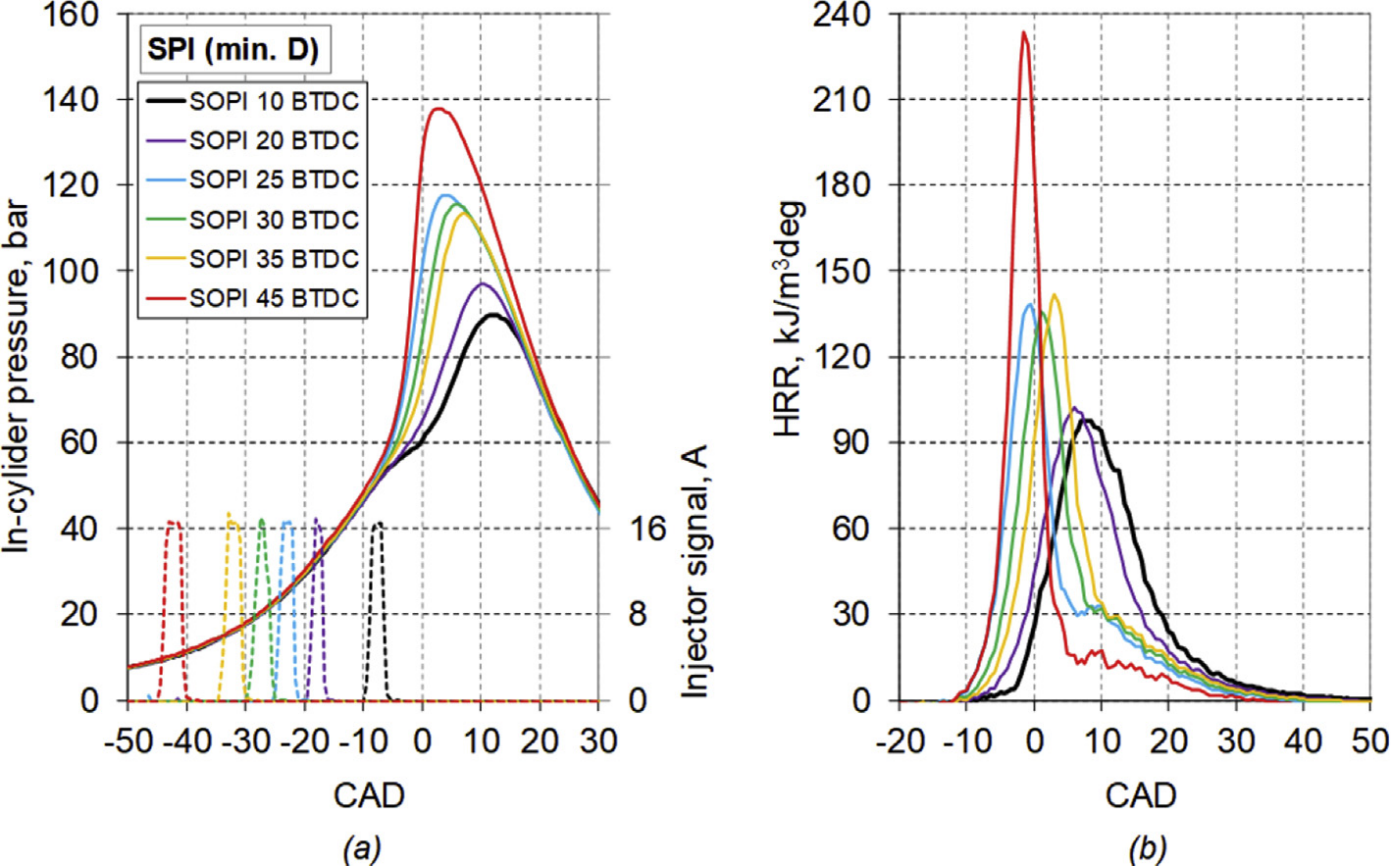
Average acquisition of *(a)* the indicated pressure cycle, diesel injector control signal and *(b)* the heat release rate curve, for the SPI case.

**Fig. 6:**
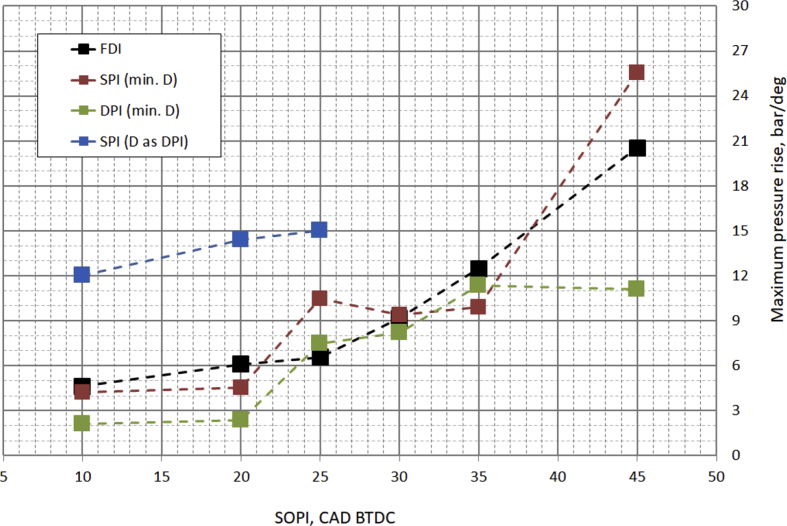
Maximum pressure rise, average over 100 cycles.

The main characteristics of the engine used for the test are listed in Table 1. Table 2 provides diesel fuel injector parameters, while Fig. 1 represents the drawing of the combustion chamber. The composition and main characteristics of natural gas used as main fuel in DF mode are reported in Table 3. Table 4 provides the specifications of the equipment used for the tests. About the test procedure: Table 5 provides the air mass flow rate and manifold pressure, while Table 6 provides the fuel flow rates and injection conditions, in all the test points. The cycle by cycle acquisitions of the intake manifold absolute pressure are available in the dataset named “Cycle-based acquisitions” of the linked public repository. The average acquisition over 100 consecutive operating cycles of the indicated pressure cycle (in one of the four cylinders), of the diesel injector control signal together with the heat release rate (HRR) curves, obtained by calculation, are plotted in Fig. 2, Fig. 3, Fig. 4, Fig. 5 respectively for the 4 cases analyzed: 1) full diesel injection (FDI), 2) DPI with minimum diesel for stable combustion, SPI 3) with the same diesel mass injected as the DPI case indicated as SPI (D as DPI) and SPI with minimum diesel for stable combustion indicated as SPI (min. D). The cycle by cycle acquisitions and calculation of these parameters are available in the dataset named “Cycle-based acquisitions” of the linked public repository. Finally, an analysis of the maximum in-cylinder pressure rise was performed and the values are plotted in Fig. 6, as average value over 100 cycles, and given in Table 7, as percentage of operating cycles affected by a certain value of the maximum pressure gradient. All the data used for that analysis, which are the values of the maximum pressure increase recorded in each of the 100 consecutive cycles acquired under the different test conditions, could easily be obtained from the individual pressure cycles available in the dataset. However, for ease of reference, they are present in the excel file called “Maximum pressure rise.xlsx” which is available in the connected public repository.

**Table 1:** Main characteristics of engine.

Type	Four-stroke
Number of cylinders	4
Displacement (cm^3^)	1910
Stroke (mm)	90.4
Bore (mm)	82
Connecting rod (mm)	145
Compression ratio	18:1
Valves/cylinder	2
Intake/Exhaust port	1 swirl/1 tumble
Intake/Exhaust valve diameter (mm)	37/36
Intake valve opening (CAD BTDC)	0
Intake valve closing (CAD ABDC)	32
Exhaust valve opening (CAD BBDC)	40
Exhaust valve closing (CAD BTDC)	2

**Table 2:** Diesel fuel injector parameters.

Injection system	Common rail
Number of nozzles	7
Nozzle diameter (mm)	0.14
Nozzle length (mm)	0.95
Volume sac (mm^3^)	0.3
Injection spray angle (°)	150
Injector axis	vertical
Flow rate @ 10 MPa pressure drop (cm^3^/min)	225

**Table 3:** Composition and main characteristics of natural gas.

Density (kg/sm^3^)	0.83
LHV (MJ/kg)	46.3
CH_4_ (% vol.)	85.4
Ethane (% vol.)	9.5
Propane (% vol.)	1.7
CO_2_ (% vol.)	1.5
N_2_ (% vol.)	1.9
H/C	3.7

**Table 4:** Instrumentation specifications for flow, pressure measurement and engine out performances.

Unit	Type	Range	Accuracy
PILOT FUEL MASS FLOW	AVL MOD 730	0–50 kg/h	±0.15% of measurement
MAIN FUEL MASS FLOW	Coriolis MICRO MOTION ELITE	0–50 kg/h	<1% of measurement
AIR FLOW	Laminar flow meter	0–350 l/s	±1% of measurement
IN-CYLINDER PRESSURE	KISTLER 6058 piezoelectric transducer	0–250 bar	±0.5% of measurement
MANIFOD PRESSURE	KISTLER 4045A5 piezoresistive transducer	0–5 bar	±0.5% of measurement
TORQUE	EDDY CURRENT DYNAMOMETER B&S FE 350-S with DS Europe load Cell	0 ÷ 1400 Nm	±0.2% of measurement
SPEED	B&S FE 350-S, pick-up	0 ÷ 8000 rpm	±1 rpm

**Table 5:** Air mass flow rate of the single cylinder and intake manifold pressure in all the test conditions.

Fueling	SOPI	Intake Manifold Pressure			Air flow rate
		minimum	average	maximum	
	CAD BTDC		bar		g/stroke
FDI	10	1.49	1.52	1.57	0.53
FDI	20	1.34	1.35	1.40	0.45
FDI	25	1.31	1.33	1.37	0.44
FDI	30	1.28	1.30	1.34	0.43
FDI	35	1.28	1.30	1.34	0.43
FDI	45	1.32	1.33	1.38	0.44
SPI (min. D)	10	1.26	1.28	1.33	0.41
SPI (min. D)	20	1.25	1.27	1.32	0.41
SPI (min. D)	25	1.24	1.26	1.31	0.40
SPI (min. D)	30	1.25	1.27	1.31	0.41
SPI (min. D)	35	1.26	1.28	1.32	0.41
SPI (min. D)	45	1.25	1.27	1.31	0.40
DPI (min. D)	10	1.35	1.37	1.42	0.44
DPI (min. D)	20	1.29	1.31	1.36	0.42
DPI (min. D)	25	1.31	1.33	1.37	0.43
DPI (min. D)	30	1.29	1.30	1.35	0.41
DPI (min. D)	35	1.27	1.29	1.34	0.41
DPI (min. D)	45	1.28	1.30	1.34	0.40
SPI (D as DPI)	10	1.23	1.25	1.30	0.39
SPI (D as DPI)	20	1.22	1.24	1.28	0.39
SPI (D as DPI)	25	1.25	1.26	1.31	0.40

**Table 6:** Injection conditions and fuel flow rates of the single cylinder.

Fueling	SOPI	SOFPI	EOFPI	SOSPI	EOSPI	SOMI	EOMI	Total diesel injection	Diesel flow rate	Gas injection	Gas flow rate
	CAD BTDC	CAD BTDC						CAD	mg/stroke	ms	mg/stroke
FDI	10	12.4	9.4			−9.0	−18.0	12.0	27		
FDI	20	20.9	17.1			−1.2	−10.4	13.1	21		
FDI	25	26.7	22.3			3.7	−5.9	14.1	21		
FDI	30	31.6	27.1			8.8	0.0	13.3	20		
FDI	35	36.6	31.4			13.8	4.2	14.8	20		
FDI	45	44.4	41.3			22.0	13.4	11.6	21		
SPI (min. D)	10	10.0	6.9					3.1	1.3	16.0	20
SPI (min. D)	20	20.0	17.0					3.0	1.1	15.8	20
SPI (min. D)	25	25.3	22.0					3.3	1.4	15.0	19
SPI (min. D)	30	29.6	26.3					3.3	1.3	15.5	19
SPI (min. D)	35	35.0	31.0					4.0	1.7	15.5	19
SPI (min. D)	45	45.0	41.0					4.0	1.7	15.5	19
DPI (min. D)	10	11.3	8.3	−10.3	−15.0			7.7	4.0	15.0	19
DPI (min. D)	20	21.0	18.0	−1.0	−6.0			8.0	3.8	13.8	17
DPI (min. D)	25	26.7	23.7	4.0	−0.6			7.6	3.8	13.7	17
DPI (min. D)	30	31.4	28.4	9.1	5.0			7.1	3.7	13.7	16
DPI (min. D)	35	36.5	33.5	14.0	10.0			7.0	3.5	13.6	16
DPI (min. D)	45	46.4	43.2	25.0	20.0			8.2	4.0	13.9	16
SPI (D as DPI)	10	10.0	5.0					5.0	3.7	13.0	15
SPI (D as DPI)	20	20.0	15.0					5.0	3.9	13.3	16
SPI (D as DPI)	25	25.6	21.0					4.6	4.0	14.5	18

**Table 7:** Analysis of the maximum pressure increase detected in the cycle.

Fueling	SOPI	Pressure gradient, bar/CAD													
		2–4	4–6	6–8	8–10	10–12	12–14	14–16	16–18	18–20	20–22	22–24	24–26	26–28	28–30
	CAD BTDC	Cycle %													
FDI	10	31	56	13											
FDI	20		56	37	7										
FDI	25		38	52	10										
FDI	30			24	50	21	5								
FDI	35				4	41	36	17	2						
FDI	45							3	15	29	18	23	10	2	
SPI (min. D)	10	36	64												
SPI (min. D)	20	36	56	8											
SPI (min. D)	25				16	84									
SPI (min. D)	30			12	59	29									
SPI (min. D)	35			4	55	37	4								
SPI (min. D)	45									4	9	25	20	22	8
DPI (min. D)	10	100													
DPI (min. D)	20	100													
DPI (min. D)	25		26	33	31	10									
DPI (min. D)	30		5	39	44	12									
DPI (min. D)	35				15	52	26	4	1						
DPI (min. D)	45				5	87	8								
SPI (D as DPI)	10				9	42	40	9							
SPI (D as DPI)	20					3	45	35	15						
SPI (D as DPI)	25					1	36	39	14	7	3				

The “Maximum pressure rise.xlsx” excel file contains all the values of the maximum pressure increase recorded in each of the 100 consecutive cycles acquired in the different test conditions.

About the “Cycle-based acquisitions” dataset, it is a folder that contains the acquisitions of in-cylinder and intake-manifold cycle pressure, diesel injector current signal and heat release rate on a crank angle basis for 100 consecutive engine running cycles in different test conditions. Each tested strategy, FDI, DPI, SPI (D as DPI), SPI (min. D) is a sub-folder of the dataset, while each test condition is a compressed file in the sub-folder. The name of each of the compressed files contains the engine test conditions (2000 rpm and 100 Nm), the injection and feeding strategy, and the nominal start angle of the diesel fuel injection. In this way it is easy to associate the other data to the file of the test: the air flow rate (available from Table 5) or the fuel flow rates (available from Table 6).

## 2 Experimental design, materials, and methods

### 2.1 Reference engine, fuels, and instrumentation

A multi-cylinder diesel engine was used, which was converted to operate in the DF mode. The engine was a light-duty four-cylinder diesel type (Table 1), with the diesel injection split into a pilot and main step and a combustion chamber as shown in Fig. 1. The diesel fuel has been direct injected at 800 bar. Main characteristics of the injectors are reported in Table 2. The diesel injector control signal was detected with a simple amperometric ring. This signal is useful to study and control the pilot diesel injection. However, the actual fuel mass flow rate is related to the control signal, but is delayed and depends on the characteristics of the injection system [3].

The DF mode was achieved by adding a timed natural gas injection system with four injectors, which introduced the fuel at 2.4 bar pressure, close to the intake valve of each cylinder, during the corresponding intake stroke. The compositions and properties of natural gas used are listed in Table 2. The engine was coupled with an eddy current dynamometer and equipped with two pressure transducers, one in the intake manifold and the other in the combustion chamber, to acquire dynamic pressures with the crank angle.

The list of instruments used, range of measurement and accuracy is given in Table 4.

### 2.2 Testing and data analysis procedures

The engine was operated at 100 Nm of brake torque and 2000 rpm. The eddy current dynamometer was controlled to maintain the engine speed constant, while two electronic modules were used to set the engine load by adapting the fuel flows. The DF operation was performed with the highest possible gaseous fuel percentage, by decreasing the diesel fuel flow rate at the minimum quantity required for a stable combustion start for the cases: DPI and SPI (min. D). For the case SPI (D as DPI), the pilot diesel fuel flow rate was set at the same amount of the DPI. The FDI case is in full diesel as reference. A set of experiments were conducted setting the start of the pilot injection (SOPI) at 10, 20, 25, 35 and 45 CAD BTDC, with the limit of not exceeding a pressure gradient of 15 bar/deg. In the cases of FDI and DPI, the second diesel fuel injection was delayed by approximately 1.7 ms compared to the first one (pilot injection).

For each test, fuel and air average mass flow rate were acquired together with in-cylinder cycle pressure (cylinder n. 3), dynamic intake-manifold pressure and diesel injector current signal, on a crank angle basis, for 100 consecutive engine running cycles.

Acquired in-cylinder pressure signal was used to perform a combustion analysis through the calculation of the net HRR which is the difference between the HRR during combustion and the heat transfer rate to the walls. HRR can be evaluated using the first law of thermodynamics [4]. HRR curves over crank angle where calculated with Equation (1), assuming constant compression/expansion polytropic coefficients α (respectively 1.37 and 1.30). In the equation, HRR is in kJ/m3deg, the pressure at each crack angle is in bar, while the instantaneous volume and the maximum cylinder volume are in m3.

(1) $$RHR_{i}=\frac{100}{\left(\alpha -1\right)\left(\theta_{i+1}-\theta_{i}\right)V_{max}}\cdot \left[\alpha \cdot p_{i}\left(V_{i+1}-V_{i-1}\right)+V_{i}\left(p_{i+1}-p_{i-1}\right)\right]$$

HRR curves have been filtered by a median filter with a window of five as in Equation (2) for removing impulsive type noise from a signal.

(2) $$RHR_{i}=\frac{1}{5}\cdot \overset{i+2}{\underset{i-2}{\sum }}RHR_{i}$$

Air mass flow rate measurement and average values of intake-manifold pressure are reported in Table 5, for all the test conditions. Table 6 provides the fuel flow rates and injection operation modes, in all the test conditions. In this last Table, SOPI is the nominal value of diesel injection start angle while the following parameters describe the injection strategies: start of the first pilot injection (SOFPI), end of the first pilot injection (EOFPI), start of the second pilot injection (SOSPI), end of the second pilot injection (EOSPI), start of the main injection (SOMI) and end of the main injection (EOMI). SOFPI and EOFPI refer to the pilot phase which is present in all the tested injection strategies, SOSPI and EOSPI refer only to the DPI strategy while SOMI and EOMI refer to main injection of the full-diesel cases.

Air and fuels flow rates per stroke in Table 5, Table 6 are relative to a single cylinder of the engine. These data were obtained by dividing by four the measured engine hourly average flow rates of air and fuels, and considering the number of intake strokes per hour, carried out by each of the four cylinders.

### 2.3 Acquisitions from the tests

The average acquisition over 100 consecutive operating cycles of the indicated pressure cycle, of the diesel injector control signal, together with the heat release rate curve, obtained by calculation, are plotted in Fig. 2, Fig. 3, Fig. 4, Fig. 5 respectively for the 4 analyzed cases: FDI, DPI with minimum diesel for stable combustion, SPI with the same diesel mass injected as the DPI case and SPI with minimum diesel.

An analysis of the maximum in-cylinder pressure rise was performed and the results are plotted in Fig. 6 as average value over 100 cycles, and given in Table 7 as percentage of operating cycles affected by a certain value of the maximum pressure gradient. In the case of SPI (D as DPI) it was not possible to test SOPI higher than 25 CAD BTDC due to a strong overcoming of the pressure gradient limit (15 bar/CAD) and the occurrence of detonation cycles.
